# Estimating range of influence in case of missing spatial data: a simulation study on binary data

**DOI:** 10.1186/1476-072X-14-1

**Published:** 2015-01-06

**Authors:** Kristine Bihrmann, Annette K Ersbøll

**Affiliations:** Faculty of Medical and Health Sciences, University of Copenhagen, Grønnegårdsvej 8, DK-1870 Frederiksberg C, Denmark; National Institute of Public Health, University of Southern Denmark, Øster Farimagsgade 5A, 2, DK-1353 Copenhagen K, Denmark

**Keywords:** Range of influence, Missing data, Binary data, INLA

## Abstract

**Background:**

The range of influence refers to the average distance between locations at which the observed outcome is no longer correlated. In many studies, missing data occur and a popular tool for handling missing data is multiple imputation. The objective of this study was to investigate how the estimated range of influence is affected when 1) the outcome is only observed at some of a given set of locations, and 2) multiple imputation is used to impute the outcome at the non-observed locations.

**Methods:**

The study was based on the simulation of missing outcomes in a complete data set. The range of influence was estimated from a logistic regression model with a spatially structured random effect, modelled by a Gaussian field. Results were evaluated by comparing estimates obtained from complete, missing, and imputed data.

**Results:**

In most simulation scenarios, the range estimates were consistent with ≤25*%* missing data. In some scenarios, however, the range estimate was affected by even a moderate number of missing observations. Multiple imputation provided a potential improvement in the range estimate with ≥50*%* missing data, but also increased the uncertainty of the estimate.

**Conclusions:**

The effect of missing observations on the estimated range of influence depended to some extent on the missing data mechanism. In general, the overall effect of missing observations was small compared to the uncertainty of the range estimate.

**Electronic supplementary material:**

The online version of this article (doi:10.1186/1476-072X-14-1) contains supplementary material, which is available to authorized users.

## Background

In spatial data, the range of influence refers to the average distance between locations at which the observed outcome is no longer correlated. The range of influence can be estimated from a variogram or based on a regression model with a spatially structured random effect, modelled by a Gaussian field. Traditionally, the regression model has posed a computational challenge in all but very small data sets, due to the need to invert a dense covariance matrix. However, the recent development of the so-called stochastic partial differential equation (SPDE) approach [1] along with the Integrated Nested Laplace Approximation (INLA) [2] approach to Bayesian inference have made these models computationally feasible. The SPDE links the Gaussian field to a Gaussian Markov random field given by a sparse precision matrix, and INLA is a computationally efficient alternative to MCMC for Bayesian inference, particularly since no sampling is required. Regression models with a spatial component could be applied in several different settings where modelling a spatial pattern is of interest. Examples include rainfall in a geographic area and pricing of houses, as well as health, disease, and lifestyle outcomes, for example the spread of an infectious disease.

Many (if not the majority of) studies are subject to missing data. For example, the price of a house is only known if the house has been sold within the study period. Similarly, all studies based on survey data will only include information on those who participated in the survey. This study will focus on the situation where a binary outcome, disease status for example, is missing at some of a given set of locations.

Missing data are often classified in three categories [3]: 1) missing completely at random (MCAR): the probability of data being missing does not depend on the observed or the unobserved data, 2) missing at random (MAR): the probability of data being missing does not depend on the unobserved data, conditional on the observed data, and 3) missing not at random (MNAR): the probability of data being missing does depend on the unobserved data, conditional on the observed data. This categorisation is based on assumptions about the missing data and cannot be tested. It is well-known that missing data can cause biased and/or inefficient estimates of, for example, regression parameters and standard errors - if not handled adequately. A popular tool for handling missing data is multiple imputation (MI) [4], at least if the missing data are not MNAR. In MI, the distribution of the observed data is used to estimate a set of values of the missing data. To our knowledge, the effect of missing data (and in particular missing outcomes) on the estimated range of influence has not been studied.

The objective of this study was to investigate how the estimated range of influence is affected when 1) a binary outcome is only observed at some of a given set of locations, and 2) multiple imputation is used to impute the outcome at the non-observed locations. For simplicity, complete information on covariates was assumed. The investigation was based on the simulation of missing data in a complete data set with known locations.

## Results and discussion

The range of influence was estimated to 12.7 km (standard deviation (SD) 4.4) in the complete data set. All the reported analyses included the only significant covariate ((log-) herd size) in the regression model. The estimation was based on a triangulation of the considered region, and a finer triangulation did not change the estimate, but did markedly increase computing time. Reducing the region to make the shape of the area more regular slightly decreased the estimated range of influence. A sensitivity analysis to determine the effect of the prior distribution of the hyperparameters showed no effect on the estimates obtained from the complete data. With missing data, the range estimate did not change when increasing the precision of the prior from 0.00001 to 0.001. In the majority of missing data scenarios, however, a precision of 0.1 produced a range estimate further from the estimate obtained from the complete data, as well as a larger standard deviation, compared to the results obtained with precision 0.001. The maximum difference in median range within scenarios was 1 km, and in most scenarios it was less than 0.5 km. These differences are small considering the uncertainty of the range estimate. They do, however, indicate that a precision of 0.1 was a potentially informative prior when the information within the data was reduced by missing observations. Therefore, a prior with precision 0.001 was preferred in the analyses. As a consequence, however, various computational problems were encountered with 75% missing data. These problems were solved by simply changing the precision to the default value of 0.1. This was done in all analyses of data sets with 75% missing data, and the results may not, therefore, be fully comparable to scenarios with fewer missing observations. The same computational problems were encountered with both 50% and 75% imputed data. In these scenarios, the precision 0.1 was also used. Overall, the variance parameter *σ*^2^ (part of the spatial covariance function) was unaffected by the prior distribution.

As part of the INLA procedure used to estimate parameters, eigenvalues of a Hessian matrix are computed. Negative eigenvalues, which may affect the accuracy of the approximation, were seen in three simulation scenarios (MAR0 OR=3, MAR1 OR=3, and MNAR OR=1/3; simulation scenarios are described in the Methods section) when 50% or more of the observations were missing. This happened in only 1 or 2 out of 1000 data sets. In imputed data, the problem was slightly more evident, especially with 75% imputed data where on average it happened in three out of 1000 data sets. Due to the limited extent, however, the problem was disregarded.

In some of the incomplete data sets, an unrealistically large estimate of the range of influence was obtained, e.g. 360 km - despite the maximum distance between locations being only 76 km. A large range estimate is the result of a small estimate of the scaling parameter *κ* in (5), which means the correlation between locations decreases slowly with increasing distance. Hence, an estimated range of influence that extended beyond the observed data was interpreted as an essentially constant correlation within the observed data, i.e. there was no detectable spatial correlation present. The situation corresponds to obtaining a flat variogram. In the overall results of the simulation study (Tables 1 and 2), data sets with an estimated range of influence larger than 75 km were excluded. Furthermore, medians (rather than means) were used to summarise the simulation results.

**Table 1:** **Summary of parameter estimates obtained from complete and simulated missing data**

Data ^***1***^	N	Intercept (SD)	RMeSE ^***2***^	Covariate ^***3***^(SD)	RMeSE ^***2***^	***σ*** ^***2***^ (SD)	RMeSE ^***2***^	Range ^***4***^ (SD)	RMeSE ^***2***^
Complete		-4.3 (0.31)	-	0.63 (0.052)	-	0.49 (0.21)	-	12.7 (4.4)	-
MCAR									
5%	1000	-4.3 (0.32)	0.047	0.62 (0.053)	0.0094	0.49 (0.21)	0.028	12.8 (4.7)	0.8
10%	1000	-4.3 (0.32)	0.066	0.62 (0.055)	0.013	0.48 (0.22)	0.039	12.9 (4.8)	1.3
15%	1000	-4.3 (0.33)	0.087	0.63 (0.056)	0.018	0.49 (0.22)	0.049	12.8 (4.9)	1.4
25%	1000	-4.3 (0.35)	0.12	0.63 (0.060)	0.025	0.47 (0.24)	0.069	13.1 (5.4)	2.0
50%	994	-4.3 (0.42)	0.21	0.63 (0.074)	0.041	0.45 (0.30)	0.13	13.1 (6.8)	3.7
75%^5^	940	-4.3 (0.56)	0.35	0.63 (0.11)	0.073	0.44 (0.48)	0.22	12.9 (10.7)	5.4
MAR0 OR=1/3									
5%	1000	-4.2 (0.32)	0.055	0.61 (0.053)	0.012	0.49 (0.21)	0.033	13.0 (4.9)	1.1
10%	1000	-4.3 (0.33)	0.078	0.63 (0.056)	0.017	0.48 (0.22)	0.042	12.6 (4.7)	1.4
15%	1000	-4.3 (0.34)	0.097	0.64 (0.058)	0.020	0.48 (0.22)	0.046	12.6 (4.8)	1.6
25%	1000	-4.3 (0.36)	0.16	0.64 (0.062)	0.028	0.50 (0.25)	0.061	13.1 (5.6)	2.1
50%	1000	-4.4 (0.44)	0.21	0.66 (0.074)	0.045	0.55 (0.32)	0.11	13.8 (6.8)	3.1
75%^*5*^	985	-4.5 (0.58)	0.33	0.67 (0.098)	0.067	0.63 (0.48)	0.20	13.4 (9.0)	4.1
MAR0 OR=3									
5%	1000	-4.1 (0.33)	0.17	0.57 (0.058)	0.048	0.47 (0.23)	0.059	13.8 (5.7)	1.7
10%	1000	-4.3 (0.33)	0.078	0.62 (0.056)	0.017	0.47 (0.22)	0.048	12.8 (5.0)	1.4
15%	1000	-4.2 (0.33)	0.10	0.62 (0.058)	0.020	0.46 (0.23)	0.062	13.1 (5.2)	1.6
25%	1000	-4.2 (0.35)	0.13	0.61 (0.062)	0.028	0.44 (0.25)	0.087	13.2 (5.8)	2.3
50%	987	-4.2 (0.41)	0.20	0.60 (0.074)	0.041	0.41 (0.31)	0.13	11.9 (7.1)	3.9
75%^5^	976	-4.2 (0.51)	0.31	0.58 (0.10)	0.066	0.35 (0.42)	0.21	10.6 (9.2)	5.3
MAR1 OR=1/3									
5%	1000	-4.7 (0.36)	0.46	0.72 (0.061)	0.093	0.50 (0.22)	0.030	12.6 (4.6)	1.0
10%	1000	-4.9 (0.38)	0.65	0.75 (0.066)	0.13	0.51 (0.23)	0.038	12.2 (4.5)	1.1
15%	1000	-5.1 (0.40)	0.80	0.78 (0.071)	0.16	0.52 (0.24)	0.047	11.8 (4.4)	1.4
25%	1000	-5.3 (0.44)	1.03	0.83 (0.079)	0.21	0.53 (0.25)	0.055	11.1 (4.1)	1.9
50%	1000	-5.6 (0.55)	1.36	0.89 (0.098)	0.27	0.58 (0.29)	0.10	9.5 (3.5)	3.3
75%^*5*^	1000	-5.9 (0.73)	1.57	0.94 (0.13)	0.31	0.61 (0.35)	0.14	9.4 (4.0)	3.5
MAR1 OR=3									
5%	1000	-4.1 (0.33)	0.17	0.57 (0.058)	0.048	0.47 (0.23)	0.059	13.8 (5.7)	1.7
10%	1000	-4.0 (0.33)	0.28	0.54 (0.062)	0.082	0.46 (0.25)	0.074	13.8 (6.1)	2.0
15%	998	-3.9 (0.34)	0.38	0.51 (0.065)	0.11	0.46 (0.26)	0.091	13.9 (6.5)	2.5
25%	985	-3.7 (0.36)	0.54	0.46 (0.072)	0.16	0.47 (0.31)	0.10	13.7 (6.7)	2.7
50%	942	-3.4 (0.40)	0.84	0.35 (0.089)	0.27	0.53 (0.43)	0.14	11.9 (7.5)	4.1
75%^*5*^	932	-3.2 (0.46)	1.10	0.25 (0.12)	0.38	0.55 (0.61)	0.22	10.3 (7.9)	4.9
MNAR OR=1/3									
5%	1000	-4.2 (0.31)	0.051	0.62 (0.052)	0.0072	0.49 (0.21)	0.019	12.8 (4.6)	0.6
10%	1000	-4.2 (0.32)	0.091	0.62 (0.053)	0.012	0.49 (0.21)	0.033	12.8 (4.7)	1.0
15%	1000	-4.1 (0.32)	0.14	0.62 (0.054)	0.014	0.49 (0.22)	0.037	12.9 (4.8)	1.2
25%	1000	-4.0 (0.33)	0.24	0.62 (0.056)	0.017	0.49 (0.23)	0.056	13.0 (5.1)	1.6
50%	997	-3.8 (0.37)	0.52	0.61 (0.065)	0.032	0.47 (0.27)	0.099	13.4 (6.1)	2.6
75%^*5*^	977	-3.4 (0.47)	0.87	0.61 (0.085)	0.047	0.45 (0.38)	0.18	12.7 (8.4)	4.2
MNAR OR=3									
5%	1000	-4.4 (0.32)	0.092	0.62 (0.055)	0.013	0.48 (0.22)	0.040	12.6 (4.7)	1.3
10%	1000	-4.5 (0.34)	0.17	0.63 (0.058)	0.023	0.48 (0.23)	0.058	12.9 (5.0)	1.7
15%	1000	-4.5 (0.35)	0.26	0.63 (0.061)	0.026	0.48 (0.24)	0.071	13.0 (5.4)	2.2
25%	999	-4.7 (0.40)	0.42	0.64 (0.068)	0.039	0.46 (0.27)	0.10	12.6 (5.7)	3.1
50%^*5*^	983	-5.0 (0.53)	0.69	0.64 (0.093)	0.068	0.44 (0.38)	0.19	12.2 (8.2)	4.9
75%^*5*^	905	-5.3 (0.83)	1.00	0.65 (0.15)	0.18	0.34 (0.62)	0.44	14.8 (19.3)	6.7

^*1*^Simulation scenarios described in the Methods section.

^*2*^Square root of the median of (est. incomplete data - est. complete data) ^2^.

^*3*^log(Herd size).

^*4*^Range of influence in km.

^*5*^Precision of prior distribution of hyperpar. changed from 0.001 to 0.1.

All results are medians of N data sets.

**Table 2:** **Summary of parameter estimates obtained after multiple imputation of simulated missing data**

Data ^***1***^	N	Intercept (SD)	RMeSE ^***2***^	Covariate ^***3***^ (SD)	RMeSE ^***2***^	***σ*** ^***2***^ (SD)	RMeSE ^***2***^	Range ^***4***^ (SD)	RMeSE ^1^
MCAR									
5%	998	-4.3 (0.32)	0.046	0.62 (0.053)	0.0095	0.49 (0.22)	0.031	13.2 (5.3)	1.0
10%	998	-4.3 (0.33)	0.071	0.62 (0.055)	0.015	0.49 (0.23)	0.042	13.4 (5.5)	1.4
15%	998	-4.3 (0.33)	0.090	0.62 (0.057)	0.019	0.48 (0.23)	0.055	13.3 (5.8)	1.6
25%	997	-4.3 (0.35)	0.12	0.63 (0.060)	0.025	0.46 (0.24)	0.077	13.2 (6.0)	2.1
50%^*5*^	924	-4.3 (0.38)	0.22	0.62 (0.066)	0.042	0.40 (0.28)	0.13	15.1 (9.2)	3.6
75%^*5*^	571	-4.2 (0.39)	0.37	0.62 (0.069)	0.069	0.44 (0.38)	0.18	15.2 (15.0)	4.2
MAR0 OR=1/3									
5%	1000	-4.3 (0.32)	0.054	0.62 (0.053)	0.012	0.49 (0.22)	0.038	13.4 (5.4)	1.3
10%	1000	-4.3 (0.33)	0.091	0.64 (0.056)	0.021	0.47 (0.22)	0.047	13.1 (5.4)	1.3
15%	1000	-4.3 (0.33)	0.10	0.64 (0.057)	0.022	0.46 (0.23)	0.058	13.1 (5.6)	1.5
25%	1000	-4.3 (0.35)	0.13	0.64 (0.060)	0.026	0.46 (0.24)	0.064	13.9 (6.4)	2.4
50%^*5*^	990	-4.3 (0.37)	0.20	0.65 (0.064)	0.041	0.42 (0.25)	0.10	14.1 (7.4)	2.7
75%^*5*^	846	-4.3 (0.39)	0.31	0.65 (0.068)	0.060	0.41 (0.30)	0.14	15.0 (9.9)	3.6
MAR0 OR=3									
5%	1000	-4.1 (0.33)	0.17	0.58 (0.056)	0.047	0.46 (0.24)	0.067	14.7 (6.7)	2.3
10%	1000	-4.2 (0.33)	0.084	0.61 (0.056)	0.018	0.47 (0.23)	0.051	13.5 (5.9)	1.6
15%	1000	-4.3 (0.33)	0.10	0.62 (0.057)	0.021	0.46 (0.25)	0.076	13.4 (6.2)	1.5
25%	986	-4.2 (0.34)	0.13	0.61 (0.059)	0.027	0.41 (0.27)	0.11	14.5 (7.3)	2.7
50%^*5*^	821	-4.0 (0.35)	0.25	0.58 (0.063)	0.047	0.36 (0.29)	0.15	14.9 (11.0)	3.8
75%^*5*^	536	-4.0 (0.37)	0.32	0.58 (0.067)	0.069	0.44 (0.52)	0.19	16.1 (20.3)	4.6
MAR1 OR=1/3									
5%	1000	-4.8 (0.34)	0.48	0.72 (0.059)	0.096	0.50 (0.23)	0.033	12.6 (5.0)	1.1
10%	1000	-4.9 (0.38)	0.61	0.75 (0.065)	0.12	0.51 (0.24)	0.041	12.4 (5.1)	1.2
15%	1000	-5.1 (0.38)	0.85	0.79 (0.066)	0.17	0.52 (0.25)	0.052	12.1 (5.0)	1.5
25%	1000	-5.4 (0.40)	1.08	0.84 (0.071)	0.22	0.53 (0.26)	0.060	11.2 (4.7)	1.9
50%^*5*^	1000	-5.6 (0.45)	1.30	0.88 (0.080)	0.26	0.54 (0.29)	0.081	10.5 (4.6)	2.5
75%^*5*^	995	-5.8 (0.51)	1.52	0.93 (0.090)	0.30	0.52 (0.32)	0.093	10.5 (5.2)	2.8
MAR1 OR=3									
5%	1000	-4.1 (0.33)	0.17	0.57 (0.055)	0.051	0.46 (0.24)	0.069	14.5 (6.7)	2.4
10%	990	-4.0 (0.33)	0.28	0.54 (0.057)	0.079	0.43 (0.25)	0.087	15.4 (8.0)	2.9
15%	934	-3.9 (0.33)	0.39	0.52 (0.058)	0.10	0.42 (0.26)	0.10	15.8 (8.7)	3.4
25%	880	-3.7 (0.34)	0.54	0.45 (0.058)	0.17	0.44 (0.30)	0.10	14.6 (8.0)	2.8
50%^*5*^	644	-3.4 (0.34)	0.85	0.36 (0.059)	0.26	0.46 (0.35)	0.14	15.7 (11.2)	3.7
75%^*5*^	566	-3.1 (0.33)	1.18	0.25 (0.062)	0.37	0.57 (0.50)	0.16	13.6 (12.6)	3.6
MNAR OR=1/3									
5%	1000	-4.2 (0.31)	0.064	0.62 (0.052)	0.0087	0.49 (0.22)	0.026	13.2 (5.3)	0.9
10%	1000	-4.2 (0.32)	0.092	0.62 (0.053)	0.011	0.49 (0.22)	0.038	13.3 (5.3)	1.2
15%	1000	-4.1 (0.32)	0.17	0.62 (0.054)	0.015	0.48 (0.22)	0.045	13.4 (5.6)	1.4
25%	1000	-4.1 (0.33)	0.22	0.62 (0.056)	0.018	0.46 (0.23)	0.064	13.7 (6.0)	1.8
50%^*5*^	972	-3.7 (0.33)	0.62	0.60 (0.057)	0.033	0.40 (0.23)	0.12	14.7 (7.3)	2.8
75%^*5*^	727	-3.3 (0.34)	0.98	0.59 (0.061)	0.051	0.34 (0.24)	0.18	15.4 (10.1)	3.9
MNAR OR=3									
5%	1000	-4.4 (0.32)	0.097	0.63 (0.055)	0.014	0.47 (0.22)	0.045	13.0 (5.3)	1.3
10%	1000	-4.5 (0.34)	0.18	0.63 (0.056)	0.023	0.49 (0.24)	0.057	13.6 (5.9)	1.8
15%	1000	-4.5 (0.40)	0.26	0.63 (0.061)	0.028	0.47 (0.25)	0.074	13.6 (6.3)	2.3
25%	983	-4.7 (0.40)	0.39	0.64 (0.067)	0.040	0.45 (0.29)	0.11	13.3 (7.0)	3.0
50%^*5*^	727	-5.0 (0.45)	0.46	0.64 (0.080)	0.046	0.50 (0.46)	0.14	13.9 (11.8)	3.6
75%^*5*^	485	-5.3 (0.55)	0.98	0.63 (0.096)	0.10	1.16 (5.5)	0.67	15.9 (27.3)	4.5

^*1*^Simulation scenarios described in the Methods section.

^*2*^Square root of the median of (est. incomplete data - est. complete data) ^2^.

^*3*^log(Herd size).

^*4*^Range of influence in km.

^*5*^Precision of prior distribution of hyperpar. changed from 0.001 to 0.1.

All results are medians of N data sets.

### Missing data

The effect of missing observations on the regression parameter estimates was dependent upon the missing data mechanism (Table 1). In the MCAR and MAR0 scenarios, the values of the estimates were not substantially affected. In the MAR1 scenarios, both parameters (intercept and covariate effect) were affected. These results were all expected, since the missing observations were dependent upon the covariate in the MAR1 scenario, whereas the missing observations did not depend upon either the covariate or the outcome in the MAR0 scenario.

In the MNAR scenarios, only the intercept was affected. The covariate effect was not affected, since only the outcome was MNAR.

The variance parameter estimates were all reasonably similar, but with a slight tendency to either increase (especially MAR0 OR=1/3 and MAR1 OR=1/3) or decrease (especially MAR0 OR=3) with more than 50% missing data. Both the standard deviation of each parameter estimate, and the Root Median Squared Error (RMeSE) increased when the number of missing observations was increased, regardless of scenario.

**Figure 1:**
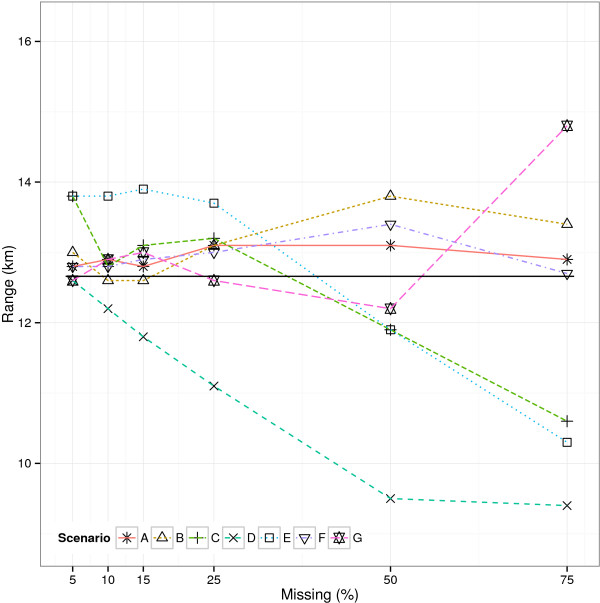
**Range of influence in missing data.** Estimated range of influence in complete data (solid line) and simulated missing data. Simulation scenarios were: A: MCAR, B: MAR0 OR=1/3, C: MAR0 OR=3, D: MAR1 OR=1/3, E: MAR1 OR=3, F: MNAR OR=1/3, G: MNAR OR=3.

The median of the estimated range of influence within each simulation scenario (Figure 1) ranged from 9.4 km (SD 4.0) (MAR1 OR=1/3, 75%) to 14.8 km (SD 19.3) (MNAR OR=3, 75%). In all scenarios except MAR1, the range estimates were quite similar with less than 50% missing observations. They tended to be slightly larger than the estimate obtained from the complete data, but differences were small, especially taking into account the uncertainty of the estimates. With ≥50*%* missing observations, the variation between scenarios increased, yet so did the standard deviation of each estimate. There was no strict pattern relating to the number of missing observations displayed, except in the MAR1 OR=1/3 scenario where the range decreased with increasing number of missing observations.

Overall, the most pronounced effect on the range estimate was seen in the MAR1 scenarios, where the missing observations were dependent upon the covariate. The specific effect of missing data on the range estimate in these scenarios is the result of the combination of the covariate and the outcome, as well as their spatial distribution, and the effect might therefore be different in another data set. The range of influence might also actually depend on the covariate, yet a potential explanation for the observed pattern is not obvious. In the MAR0 scenarios, the missing observations were directly related to the spatial structure of the data, and a more distinct effect than the observed might have been expected.

No detectable spatial correlation (range ≥ 75 km) occurred mainly among data sets with 50% and/or 75% missing observations. This is where we would expect that any spatial correlation would be most depleted by the missing data. This happened in a maximum of 95 of 1000 data sets, which was in the MNAR OR=3, 75%-scenario, where observations with a positive outcome status were most likely to be missing and hence only very reduced information about model parameters were contained in the data. The MAR1 OR=1/3 scenario was the only scenario where all data sets displayed a spatial correlation, even with 75% missing data. The MAR1 OR=3 scenario, on the contrary, had more data sets displaying no spatial correlation than any other scenario. This could partly be explained by the changed prevalence in the data (increased prevalence in the MAR1 OR=1/3 scenario and vice versa), but the pattern was not as pronounced when the missing data depended on the outcome itself (MNAR scenarios). This suggests that the observations excluded in the MAR1 OR=3 scenario exhibited the strongest spatial correlation. This would again be related to the specific data set.

The RMeSE of the range increased with an increasing number of missing observations in all scenarios. The increase was especially pronounced with more than 50% missing data. Hence, even though the overall median of the range estimates did not change much, more substantial deviations from the estimate obtained from the complete data did occur within single data sets with more than 50% missing data. In the MCAR 75% scenario, for example, the median range was 0.2 km larger than in the complete data, but the median deviation was 5.4 km.

### Imputed data

**Figure 2:**
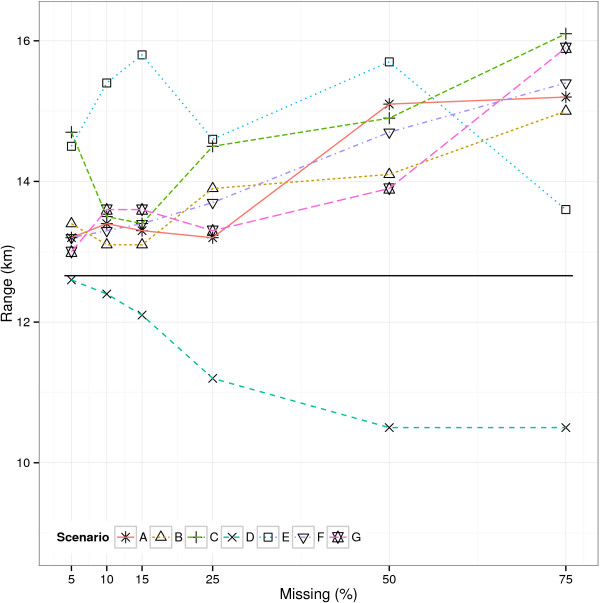
**Range of influence in multiple imputed data.** Estimated range of influence in complete data (solid line) and after multiple imputation of simulated missing data. Simulation scenarios were: A: MCAR, B: MAR0 OR=1/3, C: MAR0 OR=3, D: MAR1 OR=1/3, E: MAR1 OR=3, F: MNAR OR=1/3, G: MNAR OR=3.

Multiple imputation did not remove the bias of the regression parameter estimates introduced by the missing observations (Table 2). This was as expected, since only the outcome was missing. In that case, it is well-known that imputation will not remedy any bias of regression parameter estimates, e.g. von Hippel [5].The median of the estimated range of influence within each simulation scenario (Figure 2) ranged from 10.5 km (SD 5.2) (MAR1 OR=1/3, 75%) to 16.1 km (SD 20.3) (MAR0 OR=3, 75%). In general, the estimated range tended to be larger than the range obtained from the missing data, and hence also larger than the range obtained from the complete data set. Overall, the standard deviation of each range estimate increased after multiple imputation. With multiple imputation of less than 50% missing observations, the RMeSE tended to be slightly larger than the results obtained from the missing data. With multiple imputation of ≥ 50% missing observations, the RMeSE was slightly smaller. Therefore, considering estimation of the range of influence, at least 50% missing observations were required to potentially benefit from multiple imputation, and this was at the expense of an increased standard deviation. It should be noted, however, that the results with imputation of ≥ 50% missing observations were based on the informative prior distribution, which in case of missing data was only used with 75% missing observations.

The number of data sets (N) with detectable spatial correlation in Table 2, was not directly comparable to the corresponding number in Table 1. In multiple imputation, the incomplete data set is substituted by a set of complete data sets. If any of the data sets in such a set did not exhibit spatial correlation (i.e. the range estimate was ≥ 75 km), then the whole set was excluded from Table 2. This was done in order to retain the number of imputations in each incomplete data set. For example, 429 sets of imputed data were excluded in the MCAR 75% scenario. This means that at least 429 of 10000 imputed data sets (10 for each incomplete data set) showed no detectable spatial correlation, as compared to 60 of 1000 incomplete data sets. In this scenario actually 970 of 10000 imputed data sets showed no spatial correlation. Overall, a lack of spatial correlation occurred more frequently with imputed data than with missing data (data not shown), and mainly with imputation of more than 50% missing observations.

The parameter estimates obtained after multiple imputation without a spatial component were summarised in Table 3. Only results with imputation of 50% missing data were shown. The regression parameter results were similar to the results obtained with multiple imputation based on the spatial model. In all scenarios, the variance parameter estimate was much smaller when not including the spatial component in the imputation. The estimated range of influence also tended to be smaller, but the standard deviation of the estimate was considerable in most scenarios. Compared to imputation based on the spatial model, more data sets showed a lack of spatial correlation. This was expected, since the imputed data had no spatial structure.

**Table 3:** **Summary of parameter estimates obtained after multiple imputation of 50% simulated missing data**

Data ^***1***^	N	Intercept (SD)	RMeSE ^***2***^	Covariate ^***3***^(SD)	RMeSE ^***2***^	***σ*** ^***2***^ (SD)	RMeSE ^***2***^	Range ^***4***^(SD)	RMeSE ^2^
MCAR	732	-4.1 (0.33)	0.28	0.61 (0.062)	0.041	0.14 (0.16)	0.35	11.9 (16.3)	3.5
MAR0									
OR=1/3	963	-4.2 (0.33)	0.19	0.64 (0.064)	0.039	0.20 (0.16)	0.30	10.1 (7.4)	3.2
OR=3	628	-4.0 (0.33)	0.29	0.58 (0.063)	0.049	0.12 (0.18)	0.37	11.9 (21.3)	3.7
MAR1									
OR=1/3	998	-5.5 (0.44)	1.20	0.87 (0.079)	0.25	0.33 (0.21)	0.16	9.8 (5.3)	3.0
OR=3	625	-3.3 (0.28)	1.03	0.36 (0.057)	0.26	0.10 (0.18)	0.40	9.3 (20.3)	4.3
MNAR									
OR=1/3	772	-3.6 (0.29)	0.69	0.59 (0.054)	0.037	0.15 (0.14)	0.35	13.3 (14.1)	3.1
OR=3	636	-4.6 (0.42)	0.47	0.61 (0.078)	0.063	0.14 (0.22)	0.35	10.0 (16.2)	4.4

^*1*^Simulation scenarios described in the Methods section ^*2*^ Square root of the median of (est. incomplete data - est. complete data) ^2^ ^*3*^log(Herd size) ^*4*^Range of influence in km. Imputation was based on a standard logistic regression model without inclusion of a spatial component. All results are medians of N data sets.

## Conclusion

This simulation study investigated how the estimated range of influence was affected by missing outcomes in binary spatial data. This is a relevant topic since missing data are a common feature in many analyses. The results showed that the effect on the range estimate was to some extent dependent upon the missing data mechanism. When the missing outcomes were MCAR, MAR depending on a covariate not correlated with the outcome, or even MNAR, the range estimates were consistent with ≤25*%* missing data. When the missing outcomes were MAR depending on a covariate which correlated with the outcome, the range estimate was affected by even a moderate number of missing observations. In this specific study, however, the considered covariate was possibly also related to the range itself. This added to the complexity of the situation and may have also contributed to the effect of the missing outcomes in this scenario. In general, the overall effect of missing observations was small compared to the uncertainty of the range estimate. Multiple imputation of the missing observations provided a potential improvement in the range estimate in the case of ≥ 50% missing data, but with increased uncertainty of the estimate as a consequence.

The range of influence was estimated in a logistic regression model with a spatially structured random effect, using the recently developed INLA approach to Bayesian inference. Overall, this approach worked very efficiently. The range estimate proved to be sensitive to the prior distribution of the hyperparameters when the amount of missing observations was increased. Various computational problems sometimes encountered with many missing observations (≥50*%*), could be solved by adding prior information to the hyperparameters. Other possibilities for optimising the INLA procedure do exist and could potentially provide a better solution, especially when working with a specific data set as opposed to the automated analyses of a simulation study.

This study was based on the simulation of missing data in a specific complete data set. The “true” range of influence was defined by this complete data set and was not varied within the simulations. To fully explore a possible dependence on for example the extent of the range and the strength of the correlation, would require completely simulated data sets. This should include the spatial locations of the observations, since different spatial patterns may also influence the effect of missing observations.

## Methods

### Data

The study was based on a complete data set with simulated missing outcome. All information (outcome, covariates, and locations) was taken from the complete data set, and then some of the observations were defined to be missing, according to different simulation scenarios. Data on *Salmonella* Dublin in Danish cattle herds were used as the complete data set. These data were available, since Denmark has a mandatory surveillance program on *Salmonella* Dublin. The *Salmonella* Dublin infection as such was not of any interest in this study.

**Figure 3:**
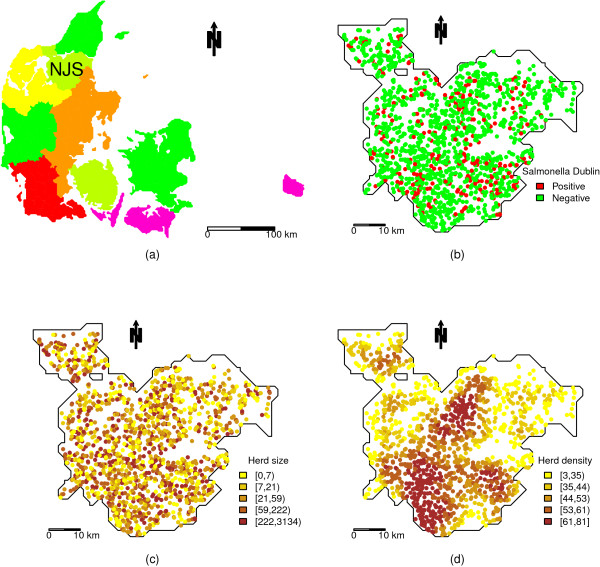
**Descriptive maps.** Denmark divided into 8 geographic regions **(a)**, including NJS (southern part of Northern Jutland) with *Salmonella* Dublin status of all cattle herds **(b)**, total number of cattle within herds **(c)**, and number of herds within a 5 km radius **(d)**.

The complete data set included all Danish cattle herds from the beginning of 2003 to the end of 2009. For all herds, information from the Danish Cattle Database (hosted by Knowledge Centre for Agriculture, Aarhus N, Denmark) included unique herd ID number, geographical coordinates in UTM-format, geographical region (Figure 3a), herd size (total number of cattle), *Salmonella* Dublin ELISA measurements on bulk-tank milk or blood samples, and date of bulk-tank milk or blood sampling. Based on this, the number of herds per km ^2^ within a 5 km radius of each herd was calculated (herd density), and all herds had a *Salmonella* Dublin classification status (positive/negative) assigned for each quarter of the year. For details on the definition of herd infection status, please refer to [6].

For the analysis, all cattle herds located in the southern part of Northern Jutland (region NJS) (Figure 3a) in the last quarter of 2008 were included (N=1597). Four herds had no information on herd size (assumed missing completely at random). Since the focus in this study is on missing outcomes, these herds were excluded; resulting in a total of N=1593 (470 dairy, 1123 non-dairy) herds. Among these, 278 herds (17.4%) had a positive *Salmonella* Dublin status (Figure 3b). The considered covariates were herd size (Figure 3c) and herd density (Figure 3d). Herd size was log-transformed since the distribution was skewed. Herd size correlated with *Salmonella* Dublin status (corr=0.35, p <0.0001), whereas herd density and *Salmonella* Dublin status did not significantly correlate (corr.=0.044, p=0.082). Herd size and herd density were uncorrelated (corr=0.035, p=0.17).

### Simulation of missing outcome

Let *y*_*i*_, *i*=1,…,1593, denote the *Salmonella* Dublin status (positive/negative) of herd *i*. Based on the completely observed data vector ***y***=(*y*_*i*_)_*i*=1,…,1593_, missing observations were generated by simulating a vector ***M***=(*M*_*i*_)_*i*=1,…,1593_ with *M*_*i*_∈{0,1}, *i*=1,…,1593. If *M*_*i*_=1, the corresponding observation *y*_*i*_ was set to missing. Scenarios with 5%, 10%, 15%, 25%, 50%, and 75% missing observations were considered. Within each scenario, 1000 replications of ***M*** were produced. Through the simulation of ***M***, observations within ***y*** were defined to be missing in three different ways: 1) missing completely at random (MCAR), 2) depending on an observed covariate (MAR), and 3) depending on the observation itself (MNAR).

Each vector ***M***=(*M*_*i*_)_*i*=1,…,1593_ was generated by drawing from independent Bernoulli distributions with parameter *π*_*i*_(= probability of being missing). To produce observations missing completely at random, *π*_*i*_ was given by
1

where *μ* was chosen corresponding to the proportion of missing data in each scenario. To produce missing observations depending on a completely observed covariate ***X***=(*X*_*i*_)_*i*=1,…,1593_, *π*_*i*_ was given by
2

Both herd size (log-transformed) and herd density were considered as the covariate ***X***. They were essentially different since herd size correlated with the outcome ***y*** (referred to as the MAR1 scenario), whereas herd density did not correlate with the outcome ***y*** (referred to as the MAR0 scenario). The parameter *μ* was chosen as above, and two values of *ν* were considered: corresponding to OR=1/3 and OR=3 of being missing when increasing the covariate one unit. Missing observations depending on the outcome ***y***=(*y*_*i*_)_*i*=1,…,1593_ were produced by letting
3

with parameters *μ*, *ν* chosen as above.

### Statistical model

Let *y*_*i*_ denote the binary outcome (0/1) at location ***z***_*i*_, *i*=1,…,*N*. With *p*_*i*_=P(*Y*_*i*_=1),*i*=1,…*N*, the logistic regression model is given by
4

where ***X***_*i*_ is a covariate (vector) with corresponding parameter (vector) ***β***, and *U*(***z***_*i*_) is a realisation of a latent stationary Gaussian field (GF) representing the spatial dependence between observations. Hence, ***U***=(*U*(***z***_*i*_))_*i*=1,…,*N*_ has a multivariate normal distribution with spatially structured covariance matrix ***Σ***. The (*r*,*s*) element of ***Σ*** is given by the Matérn spatial covariance function
5

where *Δ*_*rs*_ denotes the distance between location *z*_*r*_ and *z*_*s*_, and *K*_*λ*_ is the modified Bessel function of the second kind and order *λ*. The smoothness parameter *λ* is typically poorly identified and was fixed at 1, *κ* is a scaling parameter, and *σ*^2^ is the marginal variance. This covariance function was verified as providing a suitable model for the data by fitting it to the sample semivariogram of the residuals obtained from fitting the logistic regression (4) without the GF. Based on the covariance function (5), the range of influence is defined as , as in [1]. This corresponds to the distance at which the spatial correlation is close to 0.1 for all *λ*.

Inference about model parameters was based on the Stochastic Partial Differential Equation (SPDE) approach proposed by [1]. This approach uses a linear combination of basis functions defined on a triangulation of the spatial region to represent the GF by a Gaussian Markov random field (GMRF). Given a triangulation with *V* vertices located at  and a set of basis functions (*ψ*_*v*_)_*v*=1,…,*V*_ (each chosen to be piecewise linear with *ψ*_*v*_=1 at  and 0 at all other vertices) the GF is represented by
6

where  is a GMRF with precision matrix ***Q***(*κ*,*σ*^2^) depending on the parameters *κ* and *σ*^2^ in (5) (since *λ* is fixed at 1). Now model (4) can be rewritten as
7

where the matrix ***A***=(*A*_*iv*_(***z***_*i*_))_*i*=1,…,*N*,*v*=1,…,*V*_ is the projection from the triangulation vertices to the observation locations (which are not necessarily included as vertices).

### Inference

**Figure 4:**
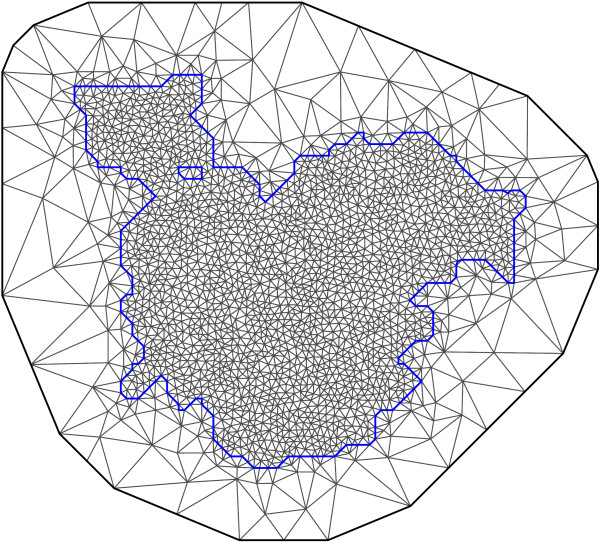
**Triangulation of the spatial region.** The mesh extends beyond the border of the considered region to correct for edge effects. The maximum allowed triangle edge length was 2 km inside the region and 50 km outside the region. The minimum allowed distance between vertices was 0.75 km. The triangulation consisted of a total of 2248 vertices.

Based on a triangulation of the spatial region and the model specified in (7), parameters were estimated using the Integrated Nested Laplace Approximation (INLA) approach proposed by [2]. This approach to Bayesian inference provides deterministic approximations to the posterior marginals for all parameters and is based on Laplace approximations [7]. Computations were done in R version 3.0.2 [8] using the INLA package (http://www.r-inla.org), which includes the SPDE approach as a standard method. The regression parameters *α*, ***β*** were assigned independent, normal prior distributions with precision 0.001, and  was assigned the GMRF with precision ***Q***(*κ*,*σ*^2^) as described above. The variance *σ*^2^ was parametrised as *σ*^2^=1/(2*πκ*^2^*τ*^2^), and the hyperparameters (log(*κ*), log(*τ*)) were assigned normal prior distributions with known precision. Sensitivity analysis to assess the effect of the prior distribution was carried out by considering three values of this precision: 0.1 (the default of the INLA package), 0.001, and 0.00001.The INLA package also provides a function for producing the required triangulation of the spatial region. The triangulation of the spatial region is shown in Figure 4. All 1593 locations were included as vertices, and additional vertices were added to produce a regular mesh. The mesh extends beyond the border of the considered region to correct for edge effects. The maximum allowed triangle edge length was 2 km inside the region and 50 km outside the region. The minimum allowed distance between vertices was 0.75 km. The triangulation consisted of a total of 2248 vertices.

To evaluate the estimates obtained in the simulation study, the Root Median Squared Error (RMeSE), alternative to the traditional Root Mean Squared Error, was calculated as the square root of the median of (estimate with complete data - estimate with missing data) ^2^ within each simulation scenario.

The simulated data were analysed using parallel computing. Analyses were run on an external supercomputing facility (i.e. a cluster of computers), due to the size of the simulation study. Parallel computing could, however, be performed on any standard personal computer with multiple CPU cores. Parallel computing is very useful for simultaneous analysis of multiple data sets, for example in simulation studies or with multiple imputed data. It cannot be used when analysing a single data set.

Parallel computing was performed using the R package parallel. With the chosen triangulation and the required output (e.g. predicted values) it took around 12-15 hours to analyse 1000 data sets (16 cores, 2.66Ghz CPU). R code is supplied as Additional file 1.

### Multiple imputation

Imputation of the simulated missing outcome was based on model (7), which was fitted to the available data. The available covariates (herd density and (log-) herd size) were included in the model. A predicted probability was sampled from the posterior distribution, and a binary outcome was then generated based on this probability. This was done at each location where the outcome was not observed, whereby a complete data set was created. This process was repeated to produce a number of imputed data sets corresponding to each incomplete data set. The number of imputed data sets created depended on the amount of missing observations. This was done in an attempt to ensure the same efficiency of the estimates across the simulation scenarios. Classical recommendations [4] suggest that only a small number of imputed data sets are needed, hence 3 data sets were created when 5% of data were missing, 5 data sets were created when 10%, 15%, 25% of data were missing, and 10 data sets were created when 50%, 75% of data were missing. Each imputed data set was analysed individually, and estimates were then combined using Rubin’s rules [3] to obtain the overall estimates corresponding to each incomplete data set. In general, each individual estimate should be approximately Gaussian distributed and otherwise transformed prior to combination [9]. The estimates of the variance parameter *σ*^2^ and the range of influence had skewed distributions, and were therefore log-transformed. The combined estimate on the original scale was subsequently obtained using standard theory for the lognormal distribution [10]. Hence, if  is the individual estimate obtained as the mean value of the log-transformed posterior distribution, and  is the combined estimate obtained from , *m*=1,…,*M*, then the combined estimate on the original scale is given by

,

where . The variance of the combined estimate on the original scale is given by

.

For comparison, the imputation model was changed from model (7) to a standard logistic regression model without inclusion of a spatial component.

## Electronic supplementary material

Additional file 1: **R code for analysis using the INLA package and parallel computing.** (TXT 4 KB)

## References

[1] Lindgren F, Rue H, Lindström J (2011). **An explicit link between Gaussian fields and Gaussian Markov random fields: the stochastic partial differentation approach**. J R Stat Soc B.

[2] Rue H, Martino S, Chopin N (2009). **Approximate Bayesian inference for latent Gaussian models by using integrated nested Laplace approximations (with discussion)**. J R Stat Soc B.

[3] Little RJA, Rubin DB (2002). Statistical analysis with missing data.

[4] Rubin DB (1987). Multiple imputation for nonresponse in surveys.

[5] von Hippel PT (2007). **Regression with missing ys: An improved strategy for analyzing multiply imputed data**. Sociol Methodol.

[6] Ersbøll AK, Nielsen LR (2008). **The range of influence between cattle herds is of importance for the local spread of*****Salmonella*****Dublin in Denmark**. Prev Vet Med.

[7] Tierney L, Kadane J (1986). **Accurate approximations for posterior moments and marginal densities**. J Am Stat Assoc.

[8] R Core Team: **R: A language and environment for statistical computing****.** Vienna, Austria: R Foundation for Statistical Computing; 2013. http://www.R-project.org/

[9] White IR, Royston P, Wood AM (2011). **Multiple imputation using chained equations: Issues and guidance for practice**. Stat Med.

[10] Aitchison A, Brown JAC (1957). The Lognormal Distribution.

